# Risk factors for parastomal hernia of loop stoma and relationships with other stoma complications in laparoscopic surgery era

**DOI:** 10.1186/s12893-020-00802-y

**Published:** 2020-06-22

**Authors:** Takuya Shiraishi, Yuji Nishizawa, Koji Ikeda, Yuichiro Tsukada, Takeshi Sasaki, Masaaki Ito

**Affiliations:** 1 Department of Colorectal Surgery, National Cancer Center Hospital East, 6-5-1 Kashiwanoha, Kashiwa, Chiba, 277-8577 Japan; 2 Department of General Surgical Science, Gunma University Graduate School of Medicine, 3-39-22 Showa-machi, Maebashi, Gunma 371-8511 Japan

**Keywords:** Laparoscopic surgery, Parastomal hernia, Peristomal skin disorders, Risk factors, Stoma-related complications

## Abstract

**Background:**

Laparoscopic approach is now a widespread technique used worldwide, but there are few recent studies on risk factors for parastomal hernia. Therefore, this study was performed to analyze the incidence of parastomal hernia in laparoscopic and open surgery in which a loop stoma was created and was intended to be temporary, and to determine risk factors for parastomal hernia formation. Associations between parastomal hernia and other stoma-related complications were also analyzed.

**Methods:**

A retrospective analysis of patient and surgical characteristics was performed in 153 consecutive patients who underwent a temporary diverting loop ileostomy or colostomy after surgery related to malignant diseases at our hospital from January to December 2016.

**Results:**

Parastomal hernia developed in 77 cases (50.3%), including 39 (25.5%) diagnosed by physical examination and 38 (24.8%) detected by CT alone. On multivariate analysis, a stoma not passing through the middle of the rectus abdominis muscle was the only independent risk factor for parastomal hernia formation (*p* = 0.005) during the median follow-up of 245.0 days. When we analyzed the factors that were associated with a stoma not passing through the middle of the rectus abdominis muscle, the only independent factor associated with this misplacement of the stoma was a laparoscopic approach (*p* = 0.012). An analysis of stoma-related complications showed that peristomal skin disorders were significantly associated with parastomal hernia (*p* = 0.049).

**Conclusions:**

This study showed that a stoma that is not formed through the middle of the rectus abdominis muscle is a risk factor for parastomal hernia formation, and that a laparoscopic approach is associated with this risk factor. Moreover, parastomal hernia is significantly associated with peristomal skin disorders.

## Background

There have been rapid advances in laparoscopic procedures for low rectal cancer, and these have increased the probability of anal preservation in patients with very low rectal cancer and reduced use of abdominal perineal resection, which does not preserve anal function [1–3]. Accordingly, there has been increased use of a temporary diverting loop stoma in patients undergoing distal colonic or rectal resection involving a distal colorectal anastomosis with risk factors for anastomotic leak related to preoperative chemoradiotherapy (CRT), male patients, and lower anastomotic level [4–6]. However, a temporary loop stoma requires another operation to close it and can also have unique complications.

Parastomal hernia is a common stoma-related complication with a reported incidence of up to 81%, and this complication induces other stoma-related complications, affects quality of life (QOL), and increases financial costs in the healthcare system [7–9]. This incidence has changed little despite advances in surgical techniques and instruments. Parastomal hernia formation following laparoscopic rectal surgery may offset the immediate benefits, such as a better cosmetic outcome and decreased postoperative pain [10, 11]. However, despite the widespread use of the laparoscopic approach worldwide, there are few recent studies on risk factors for stoma-related complications such as parastomal hernia. In addition, the position of the stoma passing through the rectus abdominis muscle and the thickness of the abdominal wall have not been investigated, although most surgeons create the stoma through the rectus abdominis muscle.

The main purpose of this study was to analyze the recent incidence of and risk factors for parastomal hernia after creation of a temporary diverting loop ileostomy or colostomy. Associations of parastomal hernia with other stoma-related complications, including peristomal skin disorders, stoma outlet obstruction, and stoma prolapse, were also evaluated.

## Methods

### Patients

The study was performed as a retrospective analysis of patients in whom stoma creation was performed at the National Cancer Center Hospital East, Japan, between January and December 2016. During this period, a stoma was created in 202 patients. After exclusion of 42 patients with an end stoma and 7 who did not undergo postoperative CT, the final study population included 153 patients who underwent a temporary diverting loop ileostomy or colostomy after surgery related to malignant diseases. This study was approved by the ethics committee of National Cancer Center Hospital (no. 2017–350).

### Data acquisition and follow-up

Data were collected for patient characteristics (sex, age, body mass index (BMI), abdominal wall thickness, rectus abdominis muscle thickness, comorbidities (diabetes mellitus (DM) or steroid use), history of open laparotomy, history of neoadjuvant chemotherapy (NAC) or preoperative CRT, emergency surgery, and American Society of Anesthesiologists (ASA) score), surgical characteristics (operation time, blood loss, blood transfusion, approach type (laparoscopy or open), stoma type (ileostomy or colostomy), specimen extraction from stoma site, stoma formed through the middle of the rectus abdominis muscle, height of stoma, incision size of stoma, base area of stoma, and volume of stoma).

**Fig. 1:**
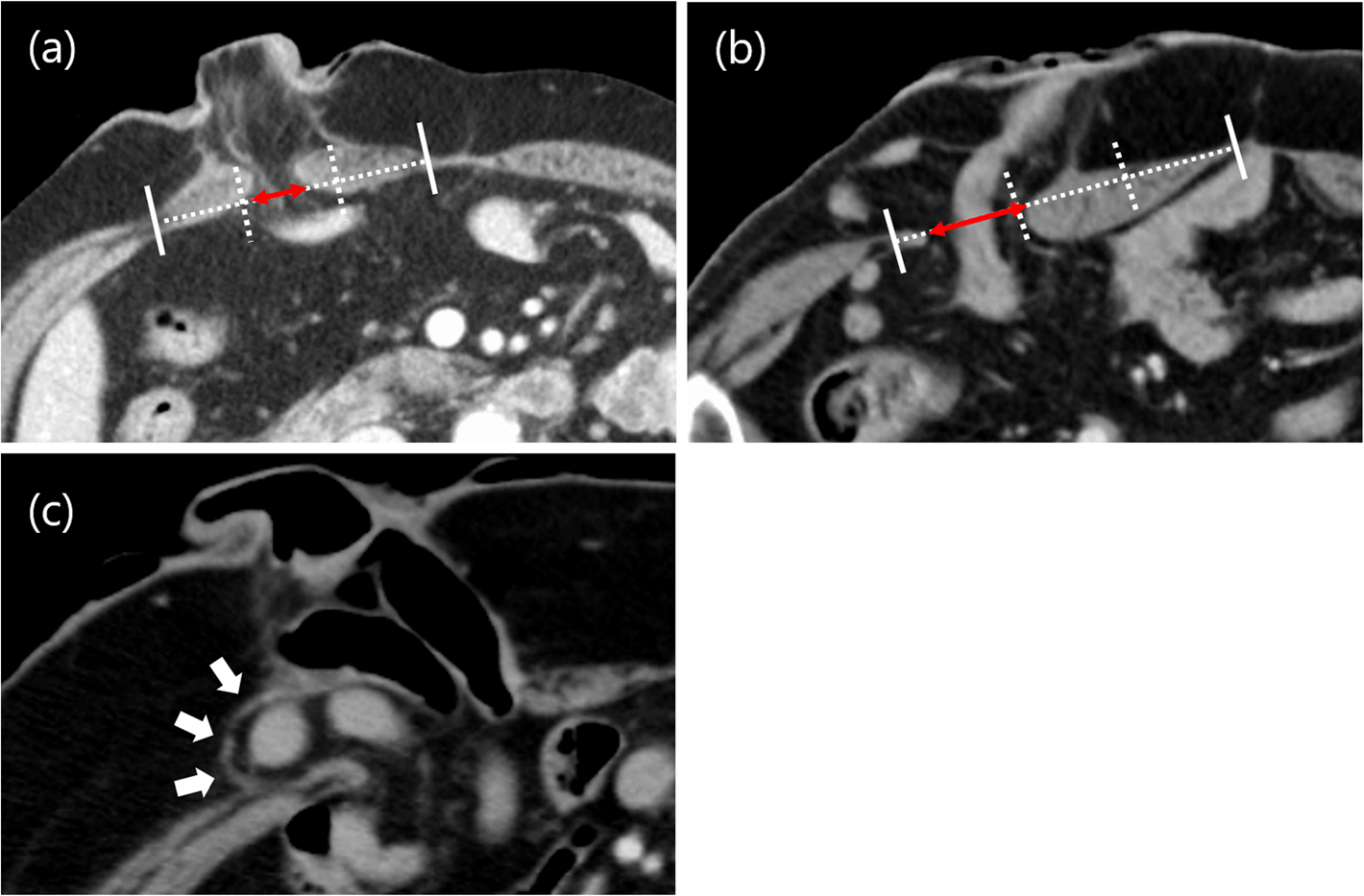
Definition of the stoma position and parastomal hernia. (**a**) A stoma passing through the middle of the rectus abdominis muscle. (**b**) A stoma not passing through the middle of the rectus abdominis muscle. (**c**) Postoperative abdominal CT was used to diagnose the parastomal hernia with a hernia sac (arrow)

The thickness of the abdominal wall and rectus abdominis muscle at the umbilical level were measured on preoperative CT by surgeons. The base area of the stoma was calculated from the vertical and horizontal size of the skin level. The stoma volume was calculated from the maximum vertical size, the maximum horizontal size, and the maximum height. These measurements of the stoma size were made postoperatively by the operating nurse. All measurements were made by a ruler. The position of the stoma through the rectus abdominis muscle was checked on postoperative CT, which was regularly performed for follow-up of malignant diseases. The rectus abdominis muscle was defined as the distance from the medial edge of the rectus abdominis muscle sheath to the lateral edge and then dividing the width of this distance into three equal parts. A stoma passing through the middle of the rectus abdominis muscle defined as one with the whole stoma passing through the center third of the rectus abdominis muscle (Fig. 1a). A stoma not passing through the middle of the rectus abdominis muscle was defined as one with the whole stoma not passing through the center third of the rectus abdominis muscle (Fig. 1b). All data were extracted from medical and operation reports. The condition of the stoma was observed during hospitalization of the patients by surgeons and wound, ostomy, and continence (WOC) nurses. Thereafter, patients were assessed during follow-up outpatient visits. For a temporary stoma, patients who did and did not receive postoperative adjuvant therapy were scheduled for stoma closure after 6 months and after 3 months, respectively. All patients were followed until closure of the stoma or until December 2017, when data were collected for the study.

### Diagnosis of parastomal hernia and other stoma-related complications

Parastomal hernia was diagnosed as any protrusion around the stoma that was detected in physical examinations by surgeons or WOC nurses. Additionally, parastomal hernia was checked on postoperative CT in a supine position, which was regularly performed for follow-up of malignant diseases. The parastomal hernia based on CT was diagnosed according to the presence of a hernia sac (Fig. 1c) [12]. The evaluation of CT examination was retrospectively reviewed by colorectal surgeon who was blinded to the patients’ clinical information. Peristomal skin disorders were diagnosed as a change in the skin, such as erythema, erosion, blister/pustule, and ulcer/tissue overgrowth. The degree of peristomal skin disorders was evaluated using the DET score. The DET score was developed by Martins et al. in 2008 as part of the Ostomy Skin Tool (OST) for evaluation of peristomal skin. The severity of discoloration (D), erosion (E), and tissue overgrowth (T) is evaluated between 0 and 2, and each area is evaluated between 0 and 3. The total DET score is the summation of severity scores and areas for D, E, and T respectively. The score is in the range from 0 to 15, where 0 represents normal skin and 15 is the worst combination of severity and extent [13, 14]. DET ≥7 was defined as a case with severe peristomal skin disorders [15]. Stoma outlet obstruction was defined was based on bowel dilation at the ostomy in clinical ileus, excluding other causes for bowel obstruction. Stoma prolapse was defined as a condition in which the full thickness of the bowel protruded through the stoma.

### Surgical techniques for stoma creation

Each stoma was preoperatively marked in the middle of the rectus abdominis muscle by surgeons and WOC nurses. According to the lifestyle of patients, the patients were marked in the best site in each of standing, sitting and supine positions. The decision to create a loop ileostomy or colostomy was made by each operating surgeon. Depending on the surgeon’s decision, a specimen was removed from a stoma site or a mini-laparotomy site. After extracting the specimen, the stoma was created using the skin bridge technique [15, 16]. The skin bridge technique uses skin instead of a plastic rod to secure and prevent retraction of the stoma. The skin bridge is made by incising the skin at the predetermined site of stoma creation, and the subcutaneous fat is divided. The skin bridge is passed through the avascular window, which is opened in the mesentery at the apex of the chosen intestinal loop, and the skin bridge is sutured to the distal edge of the opening. All stoma had a transperitoneal route and were created in the middle of the rectus abdominis muscle based on preoperative stoma site marking. All operations were performed by surgeons with substantial experience with open and laparoscopic colorectal surgery.

### Statistical analysis

Categorical variables are reported as the number (percentage) of patients, and analyzed by Chi-square or Fisher exact test, while quantitative variables are reported as a median and range, and analyzed by Mann-Whitney U test. Multivariate analysis was performed using logistic regression analysis with the backward stepwise method based on the variables in the univariate analysis required to be < 0.05 and previous reported risk factors. All statistical analyses were performed using SPSS 22.0 (SPSS Inc., Chicago, IL, USA). Significance was defined as *p* < 0.05.

## Results

Of the 153 patients, 109 were male (71.2%) and 44 were female (28.8%), the median age was 62.0 years (range 27.0–81.0 years), and the median BMI was 22.2 kg/m^2^ (range 14.7–33.4 kg/m^2^). There were 26 patients (17.0%) with DM, none were taking steroids, 48 (31.4%) had a history of open laparotomy, and 45 (29.4%) received preoperative NAC or CRT. Emergency surgery was performed in 19 patients (12.4%). A laparoscopic approach was used in 109 patients (71.2%) and an open approach in 44 (28.8%). A loop ileostomy was created in 129 patients (84.3%) and a transverse loop colostomy in 24 (15.7%). Stoma closure was performed in 124 patients (81.1%) and was not performed in 29 (18.9%) due to death or recurrence and anal incontinence. The median follow-up period was 245 days (range 47–605 days).

Parastomal hernia developed in 77 patients (50.3%), including 39 (25.5%) diagnosed by physical examination and 38 (24.8%) detected by CT alone. Potential risk factors for parastomal hernia are shown in Table 1. A stoma that did not pass through the middle of the rectus abdominis muscle was significantly associated with parastomal hernia formation in univariate analysis (*p* = 0.006). Age, BMI, abdominal wall thickness, rectus abdominis muscle thickness, DM, emergency surgery, approach type, stoma type, and specimen extraction from the stoma site were not significantly associated with parastomal hernia. In multivariate analysis, a stoma that did not pass through the middle of the rectus abdominis muscle was the only independent risk factor for parastomal hernia formation (odds ratio [OR], 2.679; 95% confidence interval [95%CI], 1.341–5.352; *p* = 0.005).

**Table 1:** Potential risk factors for parastomal hernia after creation of loop stoma

	No parastomal hernia, *n* = 76 (%)	Parastomal hernia, *n* = 77 (%)	Univariate analysis	Multivariate analysis	
			*P* value	OR (95%CI)	*P* value
Sex: male/female	52 (68)/24 (32)	57 (74)/20 (26)	0.444		
Age, years (range)	61.0 (27.0–81.0)^a^	63.0 (37.0–80.0)^a^	0.419		0.148
BMI, kg/m^2^ (range)	21.5 (14.7–31.6)^a^	23.0 (15.9–33.4)^a^	0.070		0.162
Abdominal wall thickness, mm	28.6 (12.0–50.7)^a^	30.6 (10.2–56.7)^a^	0.352		
Rectus abdominis muscle thickness, mm	10.7 (6.0–17.0)^a^	10.0 (1.0–19.0)^a^	0.239		
Diabetes mellitus	14 (18)	12 (16)	0.640		0.739
History of open laparotomy	26 (34)	22 (29)	0.452		
Preoperative treatment: NAC or CRT	19 (25)	26 (34)	0.234		
Emergency operation	11 (15)	8 (10))	0.189		
ASA score: 1/2/3	25 (33)/49 (65)/2 (3)	24 (31)/52 (68)/1 (1)	0.545		
Operation time	243.0 (19.0–840.0)^a^	290.0 (16.0–717.0)^a^	0.381		
Blood loss	48.5 (0.0–11,523.0)^a^	44.0 (0.0–3051.0)^a^	0.778		
Blood transfusion	6 (8)	5 (7)	0.737		
Approach type: laparoscopy/open	51 (67)/25 (33)	58 (75)/19 (25)	0.261		0.731
Stoma type: ileostomy/colostomy	62 (82)/14 (18)	67 (87)/10 (13)	0.355		0.604
Specimen extraction from stoma site	12 (16)	21 (27)	0.084	2.159 (0.950–4.909)	0.066
Stoma not through middle of rectus abdominis muscle	40 (53)	57 (74)	0.006	2.679 (1.341–5.352)	0.005
Height of stoma, mm (range)	11.5 (3.0–30.0)^a^	12.0 (5.0–30.0)^a^	0.163		
Incision size of stoma, mm (range)	38.0 (20.0–70.0)^a^	40.0 (20.0–71.0)^a^	0.650		0.681
Base area of stoma, cm^2^ (range)	40.6 (12.6–107.4)^a^	40.6 (18.8–93.7)^a^	0.975		
Volume of stoma, cm^3^ (range)	21.1 (4.4–110.4)^a^	22.8 (4.8–88.3)^a^	0.312		

*BMI* body mass index, *NAC* neoadjuvant chemotherapy, *CRT* chemoradiotherapy, *OR* odds ratio, *CI* confidence interval

^a^median (range)

Next, we analyzed whether there were any factors associated with a stoma not passing through the middle of the rectus abdominis muscle. We examined sex, age, BMI, abdominal wall thickness, rectus abdominus muscle thickness, emergency operation, approach type, stoma type, and specimen extraction from stoma site (Table 2). Univariate analysis showed that this kind of stoma was associated with emergency surgery (*p* = 0.044) and a laparoscopic approach (*p* = 0.011). In multivariate analysis, a laparoscopic approach was the only independent risk factor associated with misplacement of the stoma (OR, 2.522; 95%CI, 1.229–5.177; *p* = 0.012).

**Table 2:** Potential risk factors for formation of a loop stoma that does not pass through the middle of the rectus abdominis muscle

	Not through middle, *n* = 97 (%)	Through middle, *n* = 56 (%)	Univariate analysis	Multivariate analysis	
			*P* value	OR (95%CI)	*P* value
Sex: Male/Female	71 (73)/26 (27)	38 (68)/16 (32	0.482		
Age, years	63.0 (27.0–81.0)^a^	60.5 (33.0–80.0)^a^	0.459		
BMI, kg/m^2^	22.6 (14.7–33.4)^a^	21.5 (15.5–29.5)^a^	0.384		
Abdominal wall thickness, mm	30.0 (10.2–56.7)^a^	28.7 (15.6–44.9)^a^	0.299		
Rectus abdominis muscle thickness, mm					
Emergency operation	7 (7)	10 (18)	0.044		0.421
Approach type: laparoscopy/open	76 (78)/21 (22)	33 (59)/23 (41)	0.011	2.522 (1.229–5.177)	0.012
Stoma type: ileostomy/colostomy	85 (88)/12 (12)	44 (79)/12 (21)	0.138		
Specimen extraction from stoma site	20 (21)	13 (23)	0.707		

*BMI* body mass index, *OR* odds ratio, *CI* confidence interval

^a^median (range)

A total of 106 patients (69.3%) had peristomal skin disorders, including 48 (31.4%) with a DET score ≥ 7, 7 (4.6%) had stoma outlet obstruction, and 7 (4.6%) had stoma prolapse. The associations of these stoma-related complications with parastomal hernia are shown in Table 3. Peristomal skin disorders were significantly associated with parastomal hernia in univariate analysis (*p* = 0.048), and this complication retained significance in multivariate analysis (OR, 2.022; 95%CI, 1.002–4.081; *p* = 0.049).

**Table 3:** Association between parastomal hernia and other stoma-related complications after creation of loop stoma

	No parastomal hernia, *n* = 76 (%)	Parastomal hernia, *n* = 77 (%)	Univariate analysis	Multivariate analysis	
			*P* value	OR (95%CI)	*P* value
Peristomal skin disorders	47 (61)	59 (77)	0.048	2.022 (1.002–4.081)	0.049
DET score ≥ 7	23 (30)	25 (33)	0.500		
Stoma outlet obstruction	5 (7)	2 (3)	0.216		
Stoma prolapse	3 (4)	4 (5)	0.507		

*OR* odds ratio, *CI* confidence interval

## Discussion

In this study, we determined the incidence of parastomal hernia of a loop stoma in the laparoscopic era, and identified risk factors for parastomal hernia formation. We also found associations between parastomal hernia and peristomal skin disorders. An understanding of risk factors for parastomal hernia is important because this can decrease the incidence of complications, contribute to improved QOL, and reduce medical costs [7–9]. A stoma that was not formed through the middle of the rectus abdominis muscle was found to be a new risk factor for parastomal hernia, and a laparoscopic approach was associated with this risk factor. These findings provide important information for performance of optimal laparoscopic surgery.

Well-established risk factors for parastomal hernia after stoma creation include older age, increased BMI, DM, incision size, laparoscopic approach, and presence of other abdominal wall hernias [17, 18]. A stoma passing through the rectus abdominis muscle has also been reported to reduce parastomal hernia formation [18, 19]. A single study reported a lower hernia rate compared with a lateral pararectus approach, but it is uncertain whether a stoma passing through the rectus abdominis muscle prevents parastomal hernia formation, and European Hernia Society (EHS) guidelines do not indicate a preference for stoma construction at a lateral pararectus location over a transrectus location [20]. However, many surgeons recognize generally that a stoma passing through the rectus abdominis muscle reduces parastomal hernia formation, and this maneuver is performed without a clear basis in creation of a stoma.

A loop stoma that did not pass through the middle of the rectus abdominis muscle was a risk factor for parastomal hernia formation. The middle of this muscle has the greatest thickness in almost all people, and it is possible that a stoma passing through this point is protecting against parastomal hernia formation into subcutaneous fat in front of the rectus abdominis muscle. High abdominal pressure causes herniation into the weak point of the abdominal wall, including that due to operation scar, parastoma, and the inguinal canal [21]. The stoma site is generally the weakest point, but a stoma passing through the middle of the rectus abdominis muscle may have a lower incidence of parastomal hernia formation. We hypothesize that abdominal pressure might uniformly act on the parastomal site, rather than on a limited part of this site, and that this might occur more for a stoma that does not pass through the middle of the muscle. Therefore, a transrectus stoma should be created with passage through the middle of the rectus abdominis muscle.

A laparoscopic approach was found to be associated with formation of a stoma that did not pass through the middle of the rectus abdominis muscle. A laparoscopic approach has previously been identified as an independent risk factor for parastomal hernia, but no randomized trials comparing laparoscopic and open approaches have been performed [17, 22]. In stoma creation in a laparoscopic approach, especially for a temporary stoma in laparoscopic rectal surgery, the operation bed is often not flat, but in a head down and right down position, and pneumoperitoneum may remain. This position and pneumoperitoneum causes stoma site dislocation and difficulty passing the stoma through the middle of the rectus abdominis muscle because of changes in the preoperative stoma site marking and rectus abdominis position. Therefore, a laparoscopic approach may be associated with a stoma that does not pass through the middle of the rectus abdominis muscle, and might be a potential risk factor for parastomal hernia formation. Actually, we did not routinely reposition the patient flat before making the stoma in laparoscopic surgery at our institution. Despite the time required, it is important to turn the patient flat and release pneumoperitoneum before creating the site for passage of the stoma.

We also found that parastomal hernia was associated with peristomal skin disorders, as also shown in several other studies [18, 23, 24]. This may be due to frequent pouch leakage caused by parastomal hernia, which can induce damage to peristomal skin [18, 25]. However, severe peristomal skin disorders were not associated with parastomal hernia. An association between parastomal hernia and stoma prolapse has also been shown previously [26, 27], and in the present study, the incidence of stoma prolapse in cases with parastomal hernia was higher than that in cases with no parastomal hernia, but the difference was not significant. In contrast, stoma outlet obstruction of a temporary loop stoma was higher in cases with no parastomal hernia, and the fascia size has been associated with stoma outlet obstruction and parastomal hernia [25]. Thus, a shorter fascia incision results in stoma outlet obstruction, and a longer incision is linked to parastomal hernia. Thus, a low incidence of parastomal hernia might decrease peristomal skin disorders significantly, but is not related to severity, and increased stoma outlet obstruction may cause more severe problems for patients.

This study had several limitations. First, it was a retrospective study at a single institution in Japan, the number of patients was small, and there was the fairly low degree of power in analyses. Second, the follow-up period associated with hernia formation was not constant and the exact time of the onset was unclear. Time frame has been reported to be an important risk factor for parastomal hernia [18]. However, in the present study, pre- and perioperative factors, but not postoperative factors, were examined as potential risk factors for development of parastomal hernias after creation of a loop stoma. Third, the methods used for evaluating parastomal hernia might not be universally applicable. Parastomal hernia was diagnosed as any protrusion around the stoma, in addition to the parastomal hernia, based on CT. The variability in the prevalence of parastomal hernia in the literature might be due to the lack of a uniform definition. CT is the standard method for hernia diagnosis [12]. However, we believe that it is also important for patients with a protrusion around the stoma to be diagnosed clinically with parastomal hernia because this might cause stoma leakage that decrease QOL. A further analysis in a larger sample size, including cases with greater severity and using the date of diagnosis of stoma-related complications is needed to clarify the associations between risk factors and stoma-related complications, including parastomal hernia.

## Conclusion

This study revealed that a loop stoma that does not pass through the middle of the rectus abdominis muscle is a risk factor for parastomal hernia formation, and that a laparoscopic approach is associated with this risk factor. Moreover, parastomal hernia is significantly associated with peristomal skin disorders.

## Data Availability

The data that support the findings of this study are available from the ethics committee of National Cancer Center Hospital but restrictions apply to the availability of these data, which were used under license for the current study, and so are not publicly available. Data are however available from the authors upon reasonable request and with permission of the ethics committee of National Cancer Center Hospital.

## Abbreviations

CRT: Chemoradiotherapy
QOL: Quality of life
BMI: Body mass index
DM: Diabetes mellitus
NAC: Neoadjuvant chemotherapy
ASA: American Society of Anesthesiologists
WOC: Wound, ostomy, and continence
OST: Ostomy Skin Tool
OR: Odds ratio
95%CI: 95% confidence interval
EHS: European Hernia Society
